# Polyketide Synthases in the Microbiome of the Marine Sponge *Plakortis halichondrioides*: A Metagenomic Update

**DOI:** 10.3390/md12115425

**Published:** 2014-11-14

**Authors:** Gerardo Della Sala, Thomas Hochmuth, Roberta Teta, Valeria Costantino, Alfonso Mangoni

**Affiliations:** The NeaNat Group, Dipartimento di Farmacia, Università di Napoli Federico II, Via Domenico Montesano 49, 80131 Napoli, Italy; E-Mails: gerardo.dellasala@unina.it (G.D.S.); hochmuth@uni-bonn.de (T.H.); roberta.teta@unina.it (R.T.); valeria.costantino@unina.it (V.C.)

**Keywords:** marine sponges, metagenome, microbiome, PKS, polyketide synthases, Poribacteria, Porifera, protists, SupA, SwfA

## Abstract

Sponge-associated microorganisms are able to assemble the complex machinery for the production of secondary metabolites such as polyketides, the most important class of marine natural products from a drug discovery perspective. A comprehensive overview of polyketide biosynthetic genes of the sponge *Plakortis halichondrioides* and its symbionts was obtained in the present study by massively parallel 454 pyrosequencing of complex and heterogeneous PCR (Polymerase Chain Reaction) products amplified from the metagenomic DNA of a specimen of *P. halichondrioides* collected in the Caribbean Sea. This was accompanied by a survey of the bacterial diversity within the sponge. In line with previous studies, sequences belonging to *supA* and *swfA*, two widespread sponge-specific groups of polyketide synthase (PKS) genes were dominant. While they have been previously reported as belonging to Poribacteria (a novel bacterial phylum found exclusively in sponges), re-examination of current genomic sequencing data showed *supA* and *swfA* not to be present in the poribacterial genome. Several non-*supA*, non-*swfA* type-I PKS fragments were also identified. A significant portion of these fragments resembled type-I PKSs from protists, suggesting that bacteria may not be the only source of polyketides from *P. halichondrioides*, and that protistan PKSs should receive further investigation as a source of novel polyketides.

## 1. Introduction

Different species belonging to the Class *Demonspongiae* (phylum Porifera), such as the Caribbean sponge *Plakortis halichondrioides*, are associated with endosymbiotic micro-organisms, contributing to 38%–57% of the total sponge biomass [1,2]. Bacterial symbionts are mostly present in the extracellular matrix within the sponge, called the mesohyl, which contains heterotrophic (Eubacteria, Archaea) and autotrophic bacteria. As sponge-microbe interactions are widespread and some bacteria are specific and permanently associated with these sponges [3,4], the existence of sponge-bacteria symbiosis is well established. Phylogenetic analyses with sponge-derived 16S rRNA sequences revealed that sponges share a common core of bacterial communities, in spite of their taxonomical distance and their different geographical areas of origin. In addition, it has been demonstrated that some bacterial consortia are species-specific, as well as completely distinct from those inhabiting the surrounding sea water [4,5,6].

Sponge symbionts are known to produce a wide array of novel secondary metabolites of pharmaceutical interest [7]. Among these secondary metabolites, polyketides are the most important class of marine natural products from a drug discovery perspective. Several polyketides isolated from marine sponges, such as the cytotoxic compounds onnamide [8], psymberin [9], and swinholide A [10,11]) are produced by symbiotic microorganisms. Because metabolite-producing symbionts cannot be cultivated using current techniques [12], the advent of metagenomics provides an interesting and culture-independent approach to investigate the biosynthetic potential of marine sponges. The analysis of genome fragments (“genomic libraries” or “amplicon libraries”) from complex sponge-microbe consortia can lead to the isolation of the biosynthetic gene clusters of bioactive metabolites, paving the way for their large-scale, sustainable production in heterologous hosts.

Marine sponges of the genus *Plakortis* are known for the production of large amounts of polyketide peroxides, of which the antimalarial plakortin is the most abundant [13]. Our research group has extensively studied the chemistry of *P. halichondrioides* (previously identified as *P. simplex*), showing that they also contain several other unique secondary metabolites, including plakosides [14], simplexides [15], and plaxyloside [16].

*Plakortis* species are known as “high microbial abundance” (HMA) sponges [17], and this leads to the hypothesis that at least some of the secondary metabolites isolated from them are of bacterial origin. The presence in the extract of the sponge of large amounts of bacteriohopanoids, which are typical bacterial products [18], indicates that bacterial metabolism may have a high impact on the secondary metabolite pool of *Plakortis* spp., while the unique structures of some of the isolated bacteriohopanoids suggests that the metabolism of the symbiotic microbes of *Plakortis* spp. is distinct from that of the free-living microbes. Moreover, a study of the cellular localization of the metabolites typical of *P. halichondrioides* [2] further substantiates this hypothesis, in that most compounds were only present in the cells of the microbial symbionts and were not detected in the sponge cells. Finally, little is known about the biosynthesis of the polyketide peroxides or of the other characteristic metabolites of *Plakortis* spp.

The search for new biosynthetic genes in the metagenome (the collective genome of the sponge and its symbionts) of *P. halichondrioides* was undertaken by our research group some years ago, and one of the results has been the discovery of a new sponge-specific group of PKS genes, namely *swfA* [19], which has been subsequently found in several other species of sponges. Here, we report a comprehensive overview of the polyketide metabolism of *P. halichondrioides* and its symbionts based on massively parallel 454 pyrosequencing, shedding light on the existence of novel polyketide synthase pathways potentially involved in the biosynthesis of bioactive compounds, along with a survey of the diversity of bacteria associated with *P. halichondrioides*.

## 2. Results

### 2.1. Diversity of Polyketide Synthase Genes from P. halichondrioides

The metagenomic DNA of *P. halichondrioides* was used as a template for PCR amplification with, respectively, the degenerate primers KSDPQQF/KSHGTGTR [20,21] targeting highly conserved motifs in KS domains of type I PKSs, and degenerate primers AT1F/AT3R2 [19] targeting conserved motifs of acyltransferase domains of type-I PKS enzymes.

A subcloning and Sanger sequencing strategy was not judged suitable for the analysis of these complex and heterogeneous PCR amplicons. In fact, it is known that amplicons obtained in this way from metagenomes of marine sponges are almost entirely composed by sequences belonging to two widespread sponge-specific groups of PKS genes, namely *supA* [22,23] and *swfA* [19]. Therefore, massively parallel 454 pyrosequencing was performed to explore the polyketide synthase gene diversity of *P. halichondrioides* and detect novel PKS genes which are underrepresented in the amplicon mixtures and are unlikely to be detected if deep sequencing techniques are not applied.

#### 2.1.1. Phylogenetic Analysis of KS Amplicons

The 454 sequencing of the PCR products generated 19,333 reads. Of these, 1215 were shorter than 200 bp and excluded from the subsequent analysis. For 1100 (6.1%) sequences no significant BLASTx hit could be found (alignment scores were <50), and for 1466 (8.1%) sequences the BLASTx hits were not related to a KS.

As expected, BLASTx analysis of KS amplicons revealed the absolute predominance of *supA* genes, comprising over 99% of the remaining sequences. Overall, 246 non-duplicate *supA* sequences were identified and, after removal of close orthologues (dissimilarity threshold *≤*5%), at least 123 distinct *supA* variants were estimated. A remarkably high portion (15.4%) of these sequences contained one or more frameshifts. It is not known so far whether these mutations actually occur in the metagenome of *P. halichondrioides*, or whether they are PCR or sequencing artifacts.

**Figure 1:**
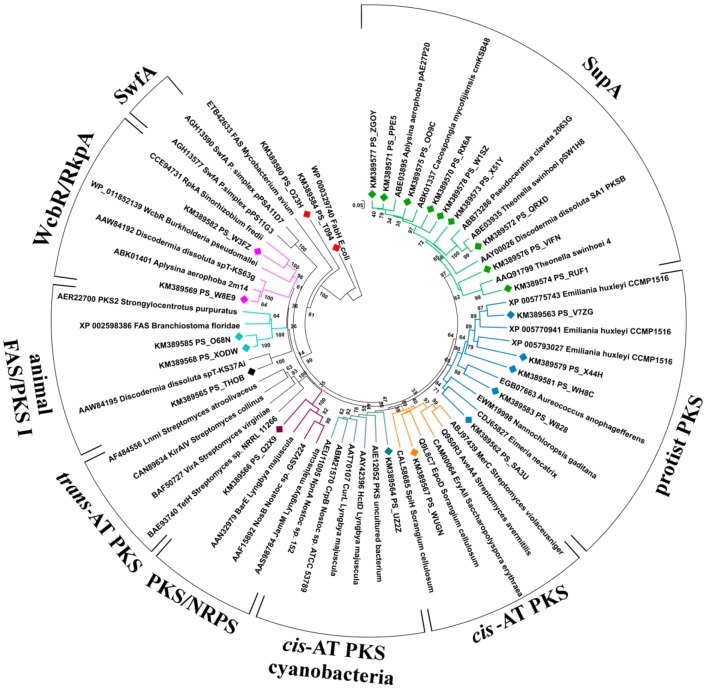
Neighbor-joining tree of KS domains from *cis*-AT PKS, *trans*-AT PKS, cyanobacterial *cis*-AT PKS, Sup, animal FAS/PKS I, protist PKS, PKS/NRPS, RkpA/WcbR, and Swf enzymes. Partial KS sequences amplified from the metagenome of *P. halichondrioides* are included in the tree and labeled with diamonds (♦). The KS tree is rooted with the *E. coli* FabH as outgroup. Bootstrap values are given at the nodes.

The non-*supA* KS fragments were limited to 21, corresponding to 15 distinct sequences after removal of duplicates and close orthologues. These non-*supA* KS sequences, a few representative *supA* sequences, and a set of ketosynthases from known PKSs extracted from GenBank were used to build a phylogenetic tree (Figure 1), which was used together with BLASTx to predict the function of the relevant PKSs.

Two KS fragments were orthologous to bacterial *wcbR/rkpA* genes, which are involved in lipopolysaccharide biosynthesis [24,25,26] (Supplementary Table S1). The BLASTx top matching sequences were KS belonging to putative *wcb*-PKS from the metagenomes of two sponges, *A. aerophoba* and *D. dissoluta*, suggesting that these KS fragments belong to sponge-specific symbionts.

Five KS fragments were related to fatty acid biosynthesis (Supplementary Table S1). Two of them (PS_T094 and PS_O23H) were orthologous with 3-oxoacyl carrier protein synthases of the type-II fatty acid synthase system of Proteobacteria, and two (PS_X0DW and PS_O68N) shared high similarity with animal type-I-PKS-like FASs. The fifth amplicon (PS_THOB, present in triplicate) found its closest homologue in the KS domain from an uncultured bacterium of the marine sponge *D. dissoluta*. While in the original paper [27] this sequence was reported to cluster with *trans*-AT PKSs, in our phylogenetic tree it appears to be more closely related to type-I-PKS-like FASs.

Besides the presence of widespread *supA*, *fas* and *wcbR/rkpA* genes, the 454 pyrosequencing method allowed the detection of eight KS fragments possibly responsible for the biosynthesis of secondary metabolites in *P. halichondrioides*. The putative functions of the respective genes, as deduced from analysis using BLASTx and the NAPDOS database [28] (which includes only PKSs with chemically characterized products from bacteria and fungi) are reported in Table 1.

**Table 1:** Selected KS fragments amplified by PCR from the metagenome of *P. halichondrioides* using degenerate primers KSDPQQF/KSHGTGTR. The putative functions of the relevant genes were deduced by *in silico* analysis using BLASTx and the NaPDos database.

Sequence	bp	G + C Content (%)	Putative KS Domain Class	BLASTx Closest Homolog (Accession#) Organism	Expect Value	Identity/Positives (% aa)	NaPDos Match
PS_Q2X9	453	64.9	PKS/NRPS	WP_004354935, *Thauera phenylacetica*	2e−76	97/98	JamM (AAS98784), *L. majuscula*
PS_WUGN	456	60.5	modular *cis*-AT	AGK63339, uncultured symbiont from *A. brasiliensis*	1e−60	70/79	EpoD (Q9L8C7), *S. cellulosum*
PS_UZ2Z	471	55.8	modular *cis*-AT	AIE12052, uncultured bacterium from mangrove soil	1e−56	60/75	CurL (AAT70107), *L. majuscula*
PS_SA3U	464	60.1	iterative PKS I	EWM19998, *Nannochloropsis gaditana*	1e−45	54/70	CALO5 (AAM70355), *M. echinospora*
PS_WH8C	465	59.4	modular *cis*-AT (starter KS)	XP_005793027, *Emiliania huxleyi* CCMP1516	1e−42	54/69	CurA (AAT70096), *L. majuscula*
PS_V7ZG	353	65.4	modular *cis*-AT	XP_005775743, *Emiliania huxleyi* CCMP1516	8e−48	74/84	MxaD (Q93TW8), *S. aurantiaca*
PS_X44H	281	52.7	modular *cis*-AT	XP_005770941, *Emiliania huxleyi* CCMP1516	5e−28	58/79	AveA4 (Q9S0R3), *S. avermitilis*
PS_W828	416	58.2	modular *cis*-AT	EGB07663, *Aureococcus anophagefferens*	1e−36	56/70	MxaB (Q93TX0), *S. aurantiaca*

The fragment PS_Q2X9 shares almost complete identity with a KS domain of a putative hybrid NRPS-PKS present in the genome of the denitrifying, aromatic-compound-degrading bacterium *Thauera phenylacetica*. The function of this NRPS-PKS is not known. The remaining seven fragments all resemble type-I PKSs (mostly *cis*-AT modular PKSs), with identity between 54% and 74%. The sequence PS_WUGN is similar to a *cis*-AT PKS fragment from the metagenome of another sponge, *Arenosclera brasiliensis*, so that its taxonomic origin is unknown, while the sequence PS_UZ2Z is related to various type-I PKS genes from the phylum *Cyanobacteria*, the BLASTx matches for the two fragments being in agreement with the phylogenetic taxonomy reported in Figure 1.

Remarkably, the closest BLASTx orthologues of all the remaining five fragments are from photosynthetic eukaryotic microorganisms (Chromista). They are grouped in a well-supported distinct clade in the KS phylogenetic tree (Figure 1), confirming the distinct phylogeny of protist PKS sequences revealed by previous studies [29,30]. The presence of a significant proportion of type-I PKSs from protists was not noted in previous studies on metagenomes of sponges, so this could be a specific characteristic of *P. halichondrioides*.

#### 2.1.2. Phylogenetic Analysis of AT Amplicons

In an attempt to overcome the overwhelming presence of *supA* genes in the KS amplicons and provide a more comprehensive view of the PKS from the metagenome of *P. halichondrioides*, a different region of type-I PKS enzymes was amplified, namely a ~280 bp region belonging to the AT domain. The degenerate primers AT1F/AT3R2 [19] were used, targeting conserved motifs of acyltransferase (AT) domains of type-I PKS enzymes. Again, the 454-pyrosequencing-method was applied to gain a detailed survey of the obtained AT fragments.

Sequencing of the PCR mixture generated 8995 reads; however, also with this modern approach, all the PKS/FAS fragments were orthologous either to *supA* or to *swfA* [19]. Almost 51% of the analyzed sequences belonged to *swfA* genes; they showed much less diversity than *supA* fragments, and only six variants related to this monomodular PKS were found, even when duplicates and close orthologues were eliminated at a dissimilarity threshold ≤1%. Only 4% of the total reads were *supA* fragments, and 20 variants orthologous to the *supA* AT group were detected. The remaining reads appeared to be unrelated to AT domains (~45% of the amplicons, Table S4). Apparently, the target amino acid motifs FPGQGsQW and QGEiAAA, recognized by the primers AT1F/AT3R2, are not specific only to PKS/FAS genes, and sequences that shared similar motifs or highly abundant sequences with less similar motifs could also be amplified by PCR.

**Figure 2:**
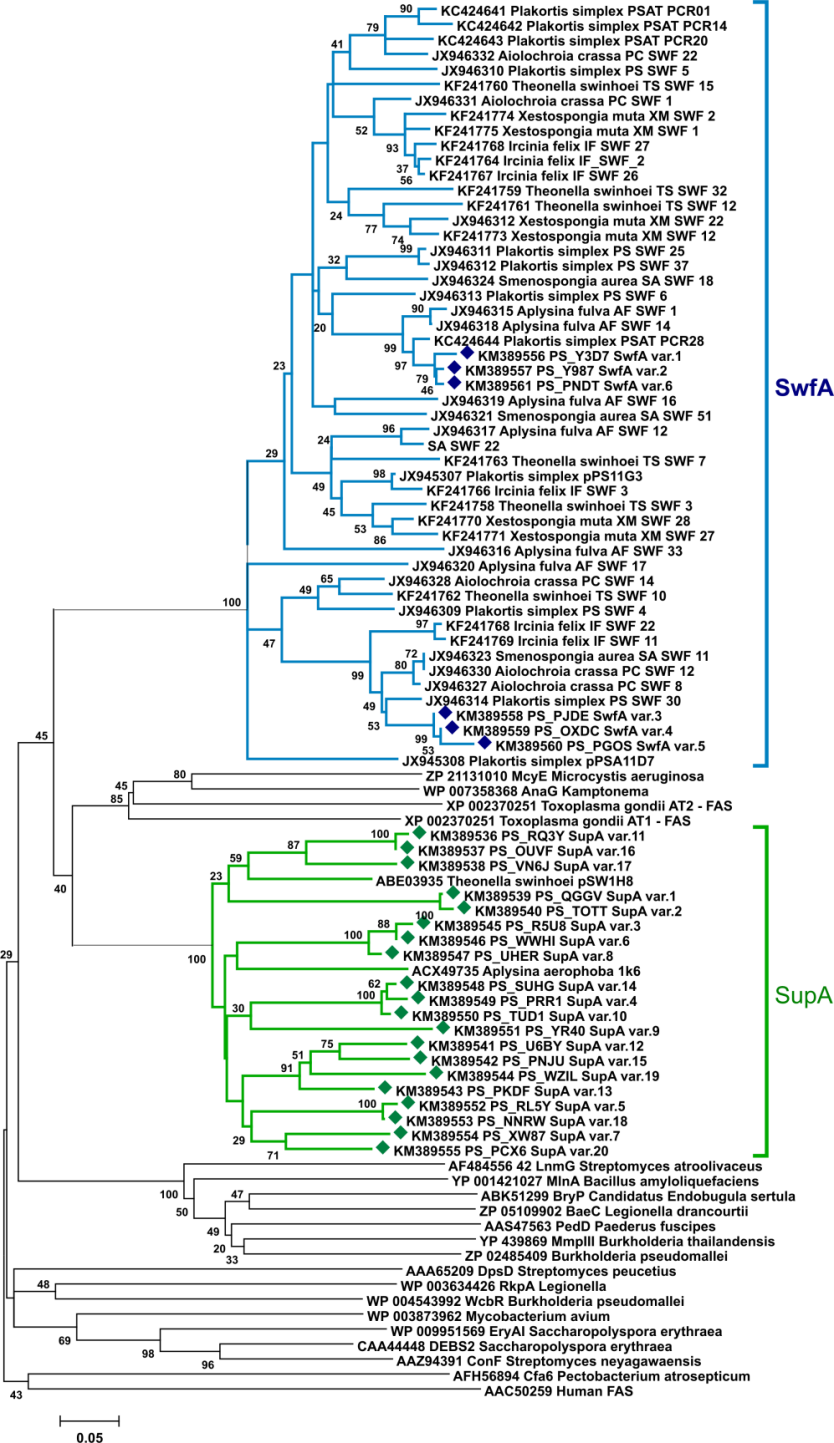
Neighbor-joining tree (p-distance model) of AT modules from SupA, SwfA, *cis-*AT PKS, *trans-*AT PKS, WcbR/RkpA, and FAS enzymes. Sup and Swf AT fragments amplified from the metagenome of *P. halichondrioides* are included in the tree and labeled with diamonds (♦). Bootstrap values are given at the nodes (values <20% are hidden).

In a phylogenetic tree where acyltransferases from PKSs of known function are included together with representative AT amplicons from *P. halichondrioides*, the formation of two distinct clades referred to the two major ubiquitous sponge PKS systems was clearly observed (Figure 2).

### 2.2. Sponge-Microbe Associations

The microbiome associated with the marine sponge *P. halichondrioides* was probed using a cultivation independent approach in order to make a preliminary survey about the identity of the sponge symbionts. A 16S rRNA gene library was prepared from metagenomic DNA extracted from sponge tissue. 16S rRNA genes were amplified by PCR using bacterial-specific 16S rRNA primers, yielding a band of the expected size of approximately 1450 bp. PCR products were subcloned via T/A cloning into the vector pBluescriptII SK (+), and 41 representative plasmids were single-read sequenced. Seven duplicate sequences were found at a dissimilarity threshold ≤2%, leading to 34 unique partial 16S rRNA gene sequences. The rRNA sequences were analysed using the RDP (Ribosomal Database Project) Seq Match tool [31] and BLASTn [32] searches, and were shown to belong for the most part to six phyla of bacteria, namely *Chloroflexi* (13 strains), *Proteobacteria* (11 strains), *Acidobacteria* (six strains), *Actinobacteria* (two strains), *Nitrospira* (one strains), *Gemmatimonadetes* (one strain).

**Figure 3:**
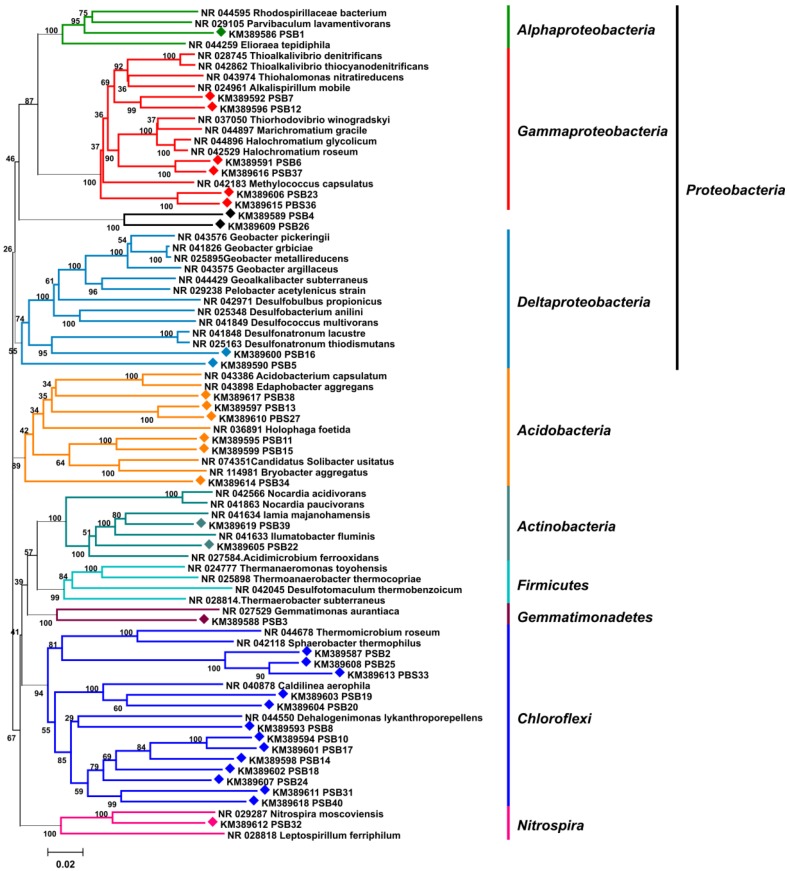
16S rRNA gene neighbor-joining phylogenetic tree displaying the taxonomy of the bacterial community associated with *P. halichondrioides.* 16S rRNA fragments amplified from the metagenome of *P. halichondrioides* are labeled with diamonds (♦). Bootstrap values are given at nodes.

None of the 41 sequences closely matched any known bacterial species, the highest sequence identity being 92% (Supplementary Table S2). Overall, the sequence analysis clearly indicated a high diversity of bacterial phylotypes in the microbiome of *P. halichondrioides*, but also a close analogy with the microbiomes of other sponges. Each strain fell in one of the sponge-specific symbiont phyla reported by Taylor *et al.* [3]. More significantly, all sequences except two were closely homologous to 16S rRNA fragments amplified from metagenomes of marine sponges, *Xestospongia muta* (FL, USA), *Aplysina cauliformis* (Belize), *Xestospongia testudinaria* (Indonesia), *Ircinia strobilina* (Bahamas) and *Geodia barretti* (Norway) being the most frequent hosts (Supplementary Table S3). A neighbor-joining tree of the 16S rRNA fragments (Figure 3), built by applying maximum composite likelihood as statistical method, displayed the same six phyla detected through the RDP Seq Match tool. The same clades and the same branching patterns were observed in minimum evolution trees using either p-distance or maximum composite likelihood statistics. Bacterial topology was supported by high bootstrap values in all phylogenetic analyses. Taken together, these data fully confirm the previous findings [3] that, irrespective of their taxonomic relationship and geographic location, marine sponges share a common, evolutionary related bacterial community.

## 3. Discussion

Previous studies on metagenomes of “high microbial abundance” (HMA) sponges [33] have shown that the vast majority of PKS genes detected through PCR screenings is represented by Sup enzymes [22], unusual, small monomodular polyketide synthases, which are widely and exclusively distributed in HMA sponges. Sup enzymes have been proposed as candidates for the biosynthesis of mid-chain-branched FAs (MBFAs), and HMA sponges are indeed one of the richest known sources for these lipids [34]. *P. halichondrioides* was not an exception to this rule, and more than 99% of the identified KS fragments belonged to *supA* genes. Remarkably, the *supA* fragments from *P. halichondrioides* revealed an extremely high diversity, and as many as 123 different sequences were identified from a single specimen of *P. halichondrioides*. A similar but lesser degree of diversity has been observed for *supA* gene fragments amplified from *Cacospongia mycofijiensis*, another HMA sponge [34].

An alternative strategy of PCR screening that attempted to overcome the overwhelming presence of *supA* fragments involved amplification of a region belonging to the AT domain of type-I PKS enzymes. This strategy effectively lowered the abundance of *supA* fragments, which comprised only 4% of the AT amplicons, and displayed only 20 variants. However, no fragments from *cis*-AT or *trans*-AT PKSs were identified among the AT amplicons. All the remaining AT fragments present in the amplicon mixture were *swfA* fragments, *i.e*., fragments belonging to the *swf* cluster, a recently discovered second family of evolutionarily distinct PKS clusters ubiquitous in HMA sponges [19].

It is worth noting that, while most of AT fragments belonged to *swfA* genes, no *swfA* sequences were found among KS fragments. This can be explained by the observation that the DPQQ motif, which is very well conserved among KS domain of type-I PKSs and was used to design the KS primers, is not present in the *swfA* genes, which therefore were not amplified among KS fragments. Because the DPQQ primers have been widely used in the previous studies on PKSs from sponge metagenomes, this explains why the widespread presence of *swfA* genes has remained unnoticed until recently.

The shallow branching topology of the *supA* and *swfA* subclades in the AT phylogenetic tree (Figure 2), which includes also fragments from other HMA sponges, suggests a common source organism for these genes. A recent report [35] seemed to have conclusively settled this problem, in that it reported the presence of both a *sup* cluster and a *swf* cluster (although annotated at that time as *wcbR*) in the genome amplified from a single cell of Candidatus Poribacteria WGA-A3 from the sponge *Aplysina aerophoba*. After this report [35], five more single-cell amplified genome sequences of Poribacteria have recently become available. Surprisingly, a BLAST search showed that none of them contains either the *sup* or the *swf* gene clusters (or, for that matter, any other type-I PKS of FAS). Because for one of them, Candidatus Poribacteria WGA-3G, the estimated genome recovery is more than 98% [36], this is unlikely due to incomplete genome coverage. At about the same time, the genome of *Candidatus Poribacteria* WGA-A3 was revised [37], and the contigs containing the *sup* and *swf* clusters are no longer present in the revised genome (apparently, they were part of the metagenome of *A. aerophoba*, but not of the genome of *Candidatus Poribacteria* WGA-A3). Therefore, there is no current evidence that the *sup* and *swf* clusters are from the genome of Poribacteria. On the contrary, it appears very likely that they are not from Poribacteria, and the identification of the source organism of these gene clusters is again an unresolved problem.

Marine sponges of the genus *Plakortis* are known for the production of very large amounts (over 25% of the total lipophilic extract) of polyketide peroxides, of which the anti-malarial plakortin [13] is the most abundant. Apart from the presence of the peroxide functional group, plakortin and its congeners are highly reduced polyketides, which are in addition characterized by the presence of ethyl branches (together with methyl branches) on the polyketide skeleton. Plakortin is likely to be biosynthesized by a type-I PKS of some symbiotic microorganism [2], and because it is present in large amounts, a highly represented type-I PKS fragment would have been expected among the PKS fragments, as well as a well-represented species of microorganism among those identified from 16S rRNA gene analysis. The results, however, were very different. First, no predominant bacterial species was detected, as each of the 34 unique 16S rRNA sequences identified was found at most in two copies. Second, in spite of the large number of amplicons sequenced, only eight putative KS fragments involved in the biosynthesis of secondary metabolites could be identified, none of which could be ascribed from sequence homology to a PKS producing an ethyl-branched, highly reduced polyketide.

The eight putative KS fragments are significantly different from each other (*E* values ≥ 10^–6^). One of them belongs to a hybrid NRPS-*cis*-AT PKS, one to an iterative type-I PKS, and six to *cis*-AT PKSs, while none belongs to a *trans*-AT PKS. Both BLASTx analyses and the rebuilt phylogenetic taxonomy revealed that they are only distantly related to PKSs of characterized function. Phylogenetic analyses suggest that two of the KS fragments from *cis*-AT PKSs are mainly related to bacterial PKSs from *Cyanobacteria*, *Actinomycetes* and *Myxobacteria*, commonly known as typical source of bioactive polyketides. It is interesting to note that only two of these fragments (PS_Q2X9 and PS_WUGN) and the *supA* fragments can clearly be classified using the KS module taxonomy proposed by Jenke-Kodama [38]. All the other KS fragments reported in this study form separate clades in this classification system (Supplementary Figure S1). These results confirm that most PKSs of sponge-associated microorganisms are evolutionarily distinct from those of free-living microorganisms [23].

Even more interestingly, the remaining five fragments resemble protist type I PKSs from the phylum *Chromista*. Type I PKSs have formerly been known only from bacteria and fungi, but recent genome sequencing has revealed the presence of type I PKSs among representatives of some protist groups [30]. Protistan type I PKS have a different architecture from those from bacteria and form a distinct clade, reflecting a different evolutionary history [29,30].

The detection of a significant proportion of putative protist type-I PKS fragments suggests the presence of protists within *P. halichondrioides*, but at present it is too early to assess whether they are symbionts or are part of the diet of *P. halichondrioides*. While symbiotic bacteria are commonly recognized as the main producers of the polyketides found in sponges, our results suggest that bacteria are not necessary the only source of polyketides from sponges. Except for polyether toxins, little is known about polyketide metabolism in protists, which deserve to be extensively investigated as sources of novel bioactive polyketides. The diversity of PKS genes is one of the most interesting aspects of polyketide metabolism in sponges, and even if these PKS genes may not produce bioactive drugs, the discovery of new PKS modules can potentially provide new modules for combinatorial polyketide synthesis via gene recombination techniques.

## 4. Experimental Section

### 4.1. Sponge Collection

The sponge *P. halichondrioides* was collected at a depth of 15 m offshore Little San Salvador Island, Caribbean Sea, Bahamas (GPS: 24°35.167' N; 75°58.476' W) during the 2010 Pawlik expedition and immediately identified onboard following the information reported on the website The Sponge Guide [39]. Individuals were cut into pieces and immediately stored in five volumes of RNA later (Life Technologies, Carlsbad, CA, USA) stabilization solution. The samples were kept at −20 °C until shipped to the laboratory, then the stabilization solution was removed and the samples were kept frozen at −80 °C until used.

### 4.2. Metagenomic DNA Isolation

700 µL of lysis buffer I (200 mM Tris-Cl, 50 mM EDTA, 1.4 M NaCl, 2% CTAB, 0.5% PVP, all in milliQ^®^-H_2_O) were added to ~40 mg of frozen sponge (in RNA later) and incubated at 37 °C for 1 h in a thermomixer (1400 rpm). After addition of 2.8 µL β-mercaptoethanol, 70 µL 10% SDS, 2 µL RNase A (100 mg/mL), and 40 µL proteinase K (10 mg/mL) the tube was incubated at 55 °C for a further hour in a thermomixer (1400 rpm). At this time, the microcentrifuge tube was spun 4 min at 5000 rpm. The clear middle phase was transferred to a new microcentrifuge tube containing 750 µL CHCl_3_ and centrifuged 10 min at 15,000 rpm. After repetition of the CHCl_3_ wash, the supernatant was transferred to a new microcentrifuge tube containing 750 µL of 70% aqueous isopropyl alcohol containing 10% (v/v) 3M NaOAc (pH 5.5) at room temperature. The precipitated DNA was spun down at top speed for 20 min, washed with ice-cold ethanol, dried and dissolved in ~60 µL elution buffer (10 mM Tris-Cl, pH 8.5).

### 4.3. Amplification of 16S rRNA Gene from P. halichondrioides

Metagenomic DNA was used for PCR amplification of 16S rRNA genes with the prokaryote specific primers F27/R1492. Polymerase chain reaction was carried out under the following conditions: initial denaturation at 94 °C for 1 min, followed by 30 cycles of 95 °C for 30 s, 48 °C for 30 s and 72 °C for 3 min, with a final extension 72 °C for 5 min. The reaction mixture (25 µL) contained: 14.65 µL H_2_O, 0.5 µL DMSO, 1.5 µL dNTP (10 mM), 2.5 µL Taq buffer advanced, 2.5 µL primer F27 (10 µM), 2.5 µL primer R1492 (10 µM), 0.35 µL RBC Taq DNA polymerase (5 U/µL, RBC Bioscience, New Taipei City, Taiwan), 0.5 µL DNA (primers: F27 5′-AGAGTTTGATCMTGGCTCAG-3′; R1492 5′-TACGGYTACCTTGTTACGACTT-3′). The PCR products were purified from the agarose gel using QIAquick gel ex kit (Qiagen, Venlo, Netherlands), subcloned via T/A cloning into pBluescriptII SK (+) (Stratagene, California, La Jolla, CA, USA), and transferred to *E. coli* XL1 Blue MRF’ (Stratagene, California, La Jolla, CA, USA) electrocompetent cells. After blue white screening plasmid preps of white colonies were digested with *Hae*III and *Msp*I for RFLP analyses. Inserts of representative plasmids were sent to GATC Biotech AG (Konstanz, Germany) for single read sequencing using the T7 primer.

### 4.4. 454 Sequencing of KS and AT Amplicons

KS and AT amplicon mixtures were obtained by PCR amplification of metagenomic DNA from *P. halichondrioides* using two primers pairs, respectively designed on the *signature regions* of type I PKSs: KSDPQQF (5'-MGN GAR GCN NWN SMN ATG GAY CCN CAR CAN MG-3') and KSHGTGR (5'-GGR TCN CCN ARN SWN GTN CCN GTN CCR TG-3') for the KS domains, AT1F (5'-TTY CCN GGN CAR GGN NSS CAG TGG-3', binding to the motif FPGQGsQW) and AT3R2 (5'-GC IGC IGC NAT CTC NCC C-3', binding to the motif QGEIAAA) for the AT domains. To obtain PCR products, 0.5 µL of sheared (by pipetting 100 times up and down) metagenomic DNA was used in a 50 µL reaction (27 µL H2O, 2 µL MgCl2 (25 mM), 3 µL DMSO, 1.5 µL dNTP (10 mM), 5 µL primers (10 mM), 5 µL Taq buffer advanced (Eppendorf, Hamburg, Germany), 1 µL RBC Taq DNA polymerase (5 U/µL, RBC Bioscience, New Taipei City, Taiwan). Three different PCR experiments with different annealing temperatures were run in parallel to make the mixture of KS amplicons as diverse as possible, and the amplicons were combined before sequencing. In case of KS primers, the PCR protocol included: initial step (45 s at 95 °C); 30 amplification cycles (denaturation: 1 min at 94 °C, annealing: 1 min at 54 °C/56 °C/58 °C, elongation: 45 s at 72 °C); final elongation (7 min at 72 °C). The AT amplicons were obtained using the same procedure and the same cycler program, except for annealing temperatures which were 58 °C, 60 °C, and 62 °C.

KS and AT PCR products were separated by gel electrophoresis, and gel extracted and purified using the QIAquick Gel Extraction Kit (Qiagen, Venlo, Netherlands). The concentrations of KS and AT amplicon libraries were adjusted to 20 ng/µL and 50 µL of both mixtures were sent to GATC Biotech AG European Genome and Diagnostics Centre (Konstanz, Germany) to be subjected to 454 pyrosequencing. Sequencing was done on a 70 × 75 FLX picotiter plate of a Roche GS FLX sequencer.

### 4.5. Bioinformatics

16S rRNA sequences were analysed using the RDP Seq Match tool [31] and BLASTn [32]. KS and AT sequences were analyzed using BLASTp and BLASTx. KS sequences were also analyzed using the NAPDOS database [28]. Alignments were performed with BioEdit [40]. Phylogenetic analyses were performed using ClustalX [41] or the MEGA 5.05 software package [42].

All the unique 16S rRNA partial sequences and representative KS and AT sequences from metagenomic analysis were deposited into GenBank under the accession numbers from KM389536 to KM389619.

## Supplementary Files

Supplementary File 1
