# Supplementary material for: Polyketide Synthases in the Microbiome of the Marine Sponge Plakortis halichondrioides: A Metagenomic Update
Source: Mar Drugs. 2014 Nov 14;12(11):5425–40. doi: 10.3390/md12115425 (PMC4245539; doi:10.3390/md12115425)

Supplementary Information

Table of Contents

Table S1 Further non-*supA* KS fragments amplified by PCR from the metagenome of *P. halichondroides*

Table S2 16S rRNA partial genes amplified from the metagenomic DNA of *P. halichondroides*, and their closest homologues in the 16S ribosomal RNA (Bacteria and Archaea) database

Table S3 Partial 16S rRNA genes amplified from the metagenomic DNA of *P. halichondroides*, and their closest homologues in the Nucleotide collection database

Table S4 Non-AT fragments (45%) amplified by PCR from the metagenome of *P. halichondrioides*

Figure S1 Neighbor-joining tree (p-distance model) obtained combining the KS sequences reported in Jenke-Kodama classification system and the KS sequences used to generate the tree in Figure 1

Table S1. Further non-*supA* KS fragments amplified by PCR from the metagenome of *P. halichondroides* using degenerate primers KSDPQQF/KSHGTGTR. The putative functions of the relevant genes were deduced by *in silico* analysis using BLASTx and the NaPDos database.

Sequence	No. of bp	Putative KS Domain Class	BLASTx Closest Homolog (accession#) Organism	Expect Value	Identity/ Positives (% aa)	NaPDos Match
PS_W3FZ	414	*wcb*-KS	AAX62362, fatty acid synthase, [bacterium H333]	6e-70	75/85	TetA (BAE93722), *S. sp. NRRL 11266*
PS_W8E9	439	*wcb*-KS	ABK01401, uncultured symbiont from *A. aerophoba*	3e-68	90/95	CALO5 (AAM70355), *M. echinospora*
PS_T094	456	KAS III	EWS78009, *Xylella fastidiosa PLS229*	6e-52	57/73	PimS3 (Q9EWA2), *S. natalensis*
PS_O23H	417	KAS I	WP_027329148, *Marinimicrobium agarilyticum*	3e-45	65/78	MegAI (Q9F830), *M. megalomicea*
PS_X0DW	451	type-I-PKS-like FAS	XP_002589000, *Branchiostoma floridae*	4e-66	69/83	LnmJ (AF484556), *S. atroolivaceus*
PS_O68N	410	type-I-PKS-like FAS	XP_002610053, *Branchiostoma floridae*	3e-50	69/81	KirAIV (CAN89634), *S. collinus*
PS_THOB	407	type-I-PKS-like FAS	AAW84195 , uncultured symbiont from *D. dissoluta*	9e-64	75/86	CurA (AAT70096), *L. majuscula*

Table S2. 16S rRNA partial genes amplified from the metagenomic DNA of *P. halichondroides*, and their closest homologues in the 16S ribosomal RNA (Bacteria and Archaea) database.

Sequence	No. of bp	BLASTn Closest Homolog (accession#) Organism	Score	Coverage/Identity (%)
PSB1	1434	NR_116475, *Magnetospira thiophila strain MMS-1*	1554	94/87
PSB2	1093	NR_102959, *Thermomicrobium roseum strain DSM 5159*	806	100/80
PSB3	1279	NR_074708, *Gemmatimonas aurantiaca strain T-27*	1271	99/85
PSB4	1527	NR_025348, *Desulfobacterium anilini strain Ani1*	1238	98/82
PSB5	1294	NR_116707, *Desulfosoma caldarium strain USBA-053*	1317	93/86
PSB6	1413	NR_112620,*Thioprofundum hispidum strain gps61*	1895	99/91
PSB7	1462	NR_112620,*Thioprofundum hispidum strain gps61*	1927	96/91
PSB8	1248	NR_074337, *Dehalogenimonas lykanthroporepellens strain BL-DC-9*	1175	99/84
PSB10	1420	NR_074397, *Caldilinea aerophila strain DSM 14535*	1197	99/82
PSB11	1395	NR_074351, *Candidatus Solibacter usitatus Ellin6076*	1592	98/88
PSB12	1517	NR_102486, *Thioalkalivibrio nitratireducens strain DSM 14787*	2115	98/92
PSB13	1476	NR_109681, *Thermoanaerobaculum aquaticum strain MP-01*	1306	94/84
PSB14	1220	NR_044550, *Dehalogenimonas lykanthroporepellens strain BL-DC-9*	1146	99/84
PSB15	1395	NR_074351, *Candidatus Solibacter usitatus Ellin6076*	1435	98/86
PSB16	1506	NR_116694, *Desulfonatronum thiosulfatophilum strain ASO4-2*	1249	99/82
PSB17	1418	NR_074337, *Dehalogenimonas lykanthroporepellens strain BL-DC-9*	1094	84/83
PSB18	1409	NR_074288, *Dehalococcoides sp. GT strain G*	1186	98/82
PSB19	1367	NR_117797, *Caldilinea tarbellica strain D1-25-10-4*	1122	98/82
PSB20	1423	NR_074397, *Caldilinea aerophila strain DSM 14535*	1568	99/87
PSB22	1486	NR_112972, *Aciditerrimonas ferrireducens strain IC-180*	1770	93/90
PSB23	1508	NR_116468, *Marichromatium gracile strain DSM 203*	1764	98/88
PSB24	1201	NR_024777, *Thermanaeromonas toyohensis strain ToBE*	1199	99/85
PSB25	1379	NR_102959, *Thermomicrobium roseum strain DSM 5159*	821	99/78
PSB26	1526	NR_075013, *Pelobacter carbinolicus strain DSM 2380*	1266	100/82
PSB27	1521	NR_109681, *Thermoanaerobaculum aquaticum strain MP-01*	1338	95/84

Table S2. *Cont.*

PSB31	1479	NR_074337, *Dehalogenimonas lykanthroporepellens strain BL-DC-9*	1197	98/82
PSB32	1503	NR_074700, *Candidatus Nitrospira defluvii*	1801	99/89
PSB33	1422	NR_102959, *Thermomicrobium roseum strain DSM 5159*	885	97/79
PSB34	1492	NR_114758, *Desulfotomaculum thermobenzoicum strain DSM 6193*	1214	84/84
PSB36	1505	NR_074692, *Thioalkalivibrio sulfidophilus strain HL-EbGR7*	1903	99/90
PSB37	1516	NR_074692, *Thioalkalivibrio sulfidophilus strain HL-EbGR7*	2030	99/91
PSB38	1531	NR_041826, *Geobacter grbiciae*	1404	99/84
PSB39	1487	NR_041634, *Iamia majanohamensis strain NBRC 102561*	1989	97/91
PSB40	1479	NR_074288, *Dehalococcoides sp. GT strain G*	1199	98/82

Table S3. Partial 16S rRNA genes amplified from the metagenomic DNA of *P. halichondroides*, and their closest homologues in the Nucleotide collection database. All sequences except two are closely homologous to 16S rRNA fragments amplified from metagenomes of marine sponges.

Sequence	No. of bp	BLASTn Closest Homolog (Accession#) Organism	Score	Coverage/Identity (%)
PSB1	1434	EF076081, Uncultured alphaproteobacterium clone PK029 from *Plakortis sp.*	2553	99/99
PSB2	1093	HE817777, Uncultured bacterium from *V. crypta*	1764	100/95
PSB3	1279	JX280155, Uncultured bacterium clone BA01-C14 from *Ircinia felix*	2329	99/99
PSB4	1527	JN596706, Uncultured deltaproteobacterium clone XD1G03 from *X. testudinaria*	2730	99/99
PSB5	1294	HQ270236, Uncultured deltaproteobacterium clone XA2D07F from *X. testudinaria*	2244	98/98
PSB6	1413	JX206582, Uncultured bacterium clone TO10-97_C6 from *I. oros*	2316	99/96
PSB7	1462	JX280173, Uncultured bacterium clone BA01-C34-seq from *I. felix*	2615	98/99
PSB8	1248	EF076074, Uncultured Chloroflexi bacterium clone PK010 from *Plakortis sp.*	2071	99/97
PSB10	1420	JQ612182, Uncultured bacterium clone GBc150 from *G. barretti*	2254	99/96
PSB11	1395	JX280180, Uncultured bacterium clone BA01-C42-seq from *I. felix*	2438	97/99
PSB12	1517	JX280259, Uncultured bacterium clone BA102-C32-seq from *I.strobilina*	2634	96/99
PSB13	1476	JX280290, Uncultured bacterium clone BA17-C27-seq from *I. felix*	2508	98/98
PSB14	1220	JN596748, Uncultured Chloroflexi bacterium clone XD2011 from *X. testudinaria*	2043	99/97
PSB15	1395	JQ612348, Uncultured bacterium clone GBc078 from *G. barretti*	1842	98/91

Table S3. *Cont.*

PSB16	1506	EF076115, Uncultured delta proteobacterium clone PK025 from *Plakortis sp.*	2689	99/99
PSB17	1418	FJ529310, Uncultured Chloroflexi bacterium clone A124 from *S. zeai*	2529	99/99
PSB18	1409	KF286150, Uncultured Chloroflexi bacterium clone BZ40D8f_f04 from *A. cauliformis*	2451	95/99
PSB19	1367	JX988646, Uncultured marine bacterium clone E12 from marine sponge	2017	99/93
PSB20	1423	JX280281, Uncultured bacterium clone BA16-C28-seq from *I.strobilina*	2492	98/99
PSB22	1486	KC669006 , Uncultured bacterium clone 14A06 from *S. pistillata*	2494	99/97
PSB23	1508	EU491139, Uncultured bacterium clone P9X2b3F06 from seafloor lavas	1829	99/89
PSB24	1201	JN596748, Uncultured Chloroflexi bacterium clone XD2011 from *X. testudinaria*	1711	97/93
PSB25	1379	HE817777, Uncultured bacterium from *V. crypta*	2065	99/93
PSB26	1526	JN596706, Uncultured deltaproteobacterium clone XD1G03 from *X. testudinaria*	1810	95/89
PSB27	1521	GU118535, Uncultured bacterium clone Mfav_D24 from *M. faveolata*	2678	97/99
PSB31	1479	JX206718 , Uncultured bacterium clone TV10-912_C6 from *I. variabilis*	2628	98/99
PSB32	1503	HQ270256, Uncultured Nitrospira sp. clone XA3B05F from *X. testudinaria*	2708	99/99
PSB33	1422	HE817777, Uncultured bacterium from *V. crypta*	2362	99/96
PSB34	1492	FJ269262 ,Uncultured Acidobacteria bacterium clone XA1G11F from *X. testudinaria*	2656	99/99
PSB36	1505	EU491139, Uncultured bacterium clone P9X2b3F06 from seafloor lavas	2045	99/91
PSB37	1516	HQ270232, Uncultured gamma proteobacterium clone XA2C10F from *X. testudinaria*	2691	99/99
PSB38	1531	FJ269286, Uncultured Acidobacteria bacterium clone XA2H05F from *X. testudinaria*	2758	99/99
PSB39	1487	JQ612226, Uncultured bacterium clone GBc022 from *G. barretti*	2606	99/99
PSB40	1479	JQ612191, Uncultured bacterium clone GBc092 from *G. barretti*	2555	98/98

Table S4. Non-AT fragments (45%) amplified by PCR from the metagenome of *P. halichondrioides* using degenerate primers AT1F/AT3R2. The putative functions of the relevant genes were deduced by *in silico* analysis using BLASTx database. Sequences shorter than 100 bp and/or occurring in ≤6 copies in the amplicon mixture (~10%) were not reported in the following table.

No of Sequences (%)	Putative Function
1729 (19.3)	unknown (blastx alignment score < 50 and/or no BLASTx hits at all)
343 (3.83)	adenylosuccinate synthetase
295 (3.29)	acetyl-/propionyl-CoA carboxylase carboxyltransferase (ACC/PCC)
116 (1.30)	hydrolases (incl. 15 dihydroorotases, 47 peptidases (incl. 14 amidases), 10 sulfatases)
108 (1.21)	WD-40 repeat containing protein
93 (1.04)	transposons/retrotransposons
84 (0.94)	Tpr repeat protein
77 (0.86)	DUF1802
66 (0.74)	kinases
61 (0.68)	oxidoreductases (incl. 26 sequences of dehydrogenases)
59 (0.66)	ABC transporter/antiporter
29 (0.32)	hydroxylases/oxygenases
28 (0.31)	SwfA, not intact
16 (0.18)	EngA (GTP-binding protein)
15 (0.17)	DNA/RNA polymerase
13 (0.14)	dehydratases
10 (0.11)	SupA, not intact
8 (0.09)	von Willebrand factor type A
6 (0.07)	ligases (other than adenylosuccinate synthetases and ACC/PCC)

Figure S1. Neighbor-joining tree (p-distance model) obtained combining the KS sequences reported in Jenke-Kodama classification system and the KS sequences used to generate the tree in Figure 1. Partial KS sequences amplified from the metagenome of *P. halichondrioides* are included in the tree and labeled with diamonds (♦). Bootstrap values are given at the nodes.

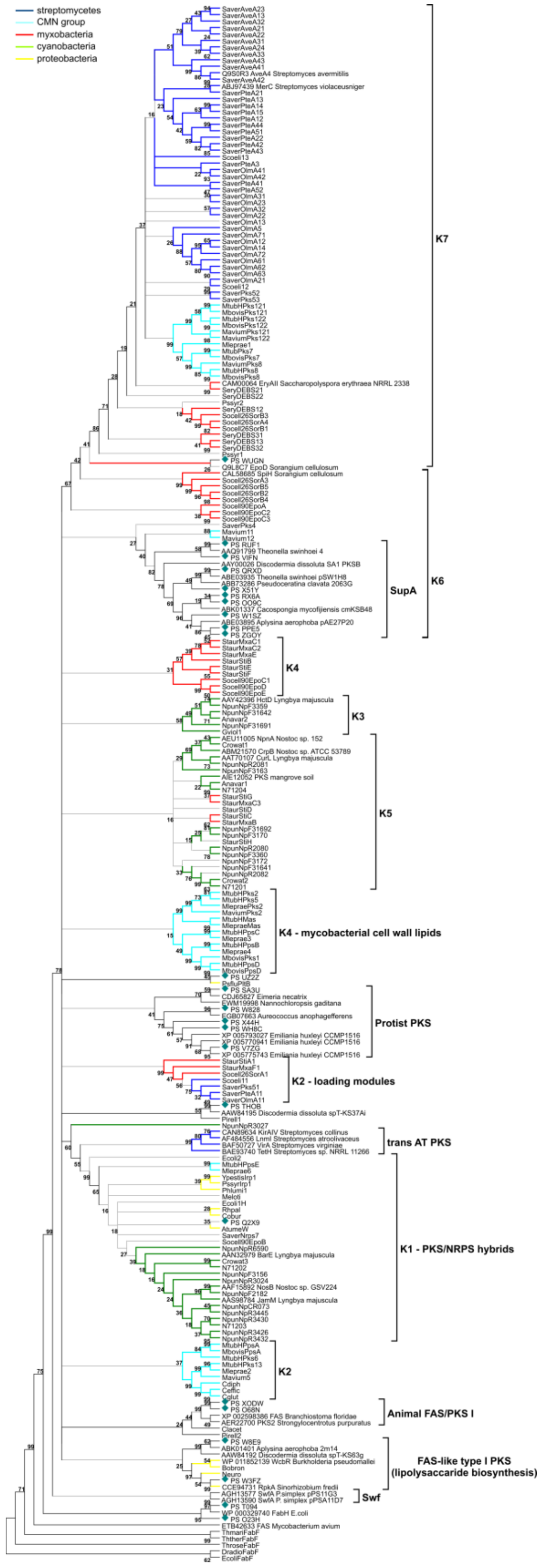

Supplement: Supplementary File 1 [file marinedrugs-12-05425-s001.pdf]
